# Elucidating the role of fatty acid reprogramming in ovarian cancer: insights cross-talk between blood, subcutaneous fat, and ovarian cancer tissues

**DOI:** 10.3389/fonc.2025.1530487

**Published:** 2025-04-30

**Authors:** Xiaocui Zhong, Mamona Bilal, Yanqiu Zhou, Xiaojia Yang, Zuchao Qin, Qibing Li, Yang Yang, Ting-Li Han, Min Li

**Affiliations:** ^1^ Department of Obstetrics and Gynaecology, The Second Affiliated Hospital of Chongqing Medical University, Chongqing, China; ^2^ Department of Occupational and Environmental Hygiene, School of Public Health and Management, Research Center for Medicine and Social Development, Innovation Center for Social Risk Governance in Health, Chongqing Medical University, Chongqing, China; ^3^ Department of Obstetrics, The First Affiliated Hospital of Chongqing Medical University, Chongqing, China; ^4^ Liggins Institute, The University of Auckland, Auckland, New Zealand

**Keywords:** ovarian cancer, fatty acid reprogramming, ovarian tissue, subcutaneous fat tissue, plasma, tumor progression

## Abstract

**Introduction:**

Aberrant fatty acid (FA) metabolism is increasingly recognized as a significant factor in ovarian cancer (OC) progression, although the comprehensive metabolic alterations across different body tissues remain unclear.

**Methods:**

In this study, sixteen OC patients and twenty-nine non-cancer (NC) patients were recruited for metabolic profiling using a global and targeted metabolomic strategy based on a gas chromatography-hydrogen flame ionization detector (GC-FID). The patient survival was followed up to 3 years, and PFS was calculated.

**Results:**

Our findings revealed distinct metabolite profiles that differentiate OC from NC groups across all sample types. We found seven, nine, and thirteen significant metabolites in subcutaneous fat, plasma, and ovarian tissue respectively. In particular, docosahexaenoic acid (DHA) and arachidonic acid (AA) levels were notably elevated in all sample types of OC patients. Furthermore, receiver operating characteristic (ROC) analysis highlight that three plasma FA showed the best specificity and sensitivity in differentiating the OC group from the NC group (Area Under The Curve, AUC > 0.89), including caprylic acid, myristoleic acid, and tetracosaenoic acid. Most of the significant FA in subcutaneous fat and ovarian tissue showed a high risk of OC. However, caprylic acid and tetracosanoic acid were identified as protective factors in the plasma sample. We also found that high levels of linoelaidic acid in subcutaneous fat and palmitelaidic acid in ovarian tissue were associated with poor prognosis. Pathway analysis indicated upregulation of fatty acid synthesis, inflammatory signaling, and ferroptosis pathways in OC patients.

**Discussion:**

This study reveals a coordinated reprogramming of FA metabolism across multiple biospecimens in OC patients. Our results suggest that specific fatty acids may contribute to OC progression through dysregulation of fatty acid synthesis, inflammatory signaling, and ferroptosis. These findings offer mechanistic insights into OC progression and highlighting potential biomarkers and targeted therapeutic interventions.

## 1 Introduction

Ovarian cancer (OC) remains a persistent public health concern globally, with significant impacts on morbidity and mortality (1). OC ranks as the seventh most commonly diagnosed cancer among women worldwide and the eighth leading cause of cancer-related deaths in 2023 (2, 3). In china, OC has emerged as the second leading cause of death among gynecological cancer (4). In 2019, there were approximately 196,000 cases of OC in China, with 45,000 new cases and 29,000 deaths attributed to this cancer (5). The age-standardized rates (ASRs) of OC prevalence, incidence and mortality have increased by 105.98%, 79.19%, and 58.93%, respectively, from 1990 to 2021 (6). 70% of OC patients were diagnosed at an advanced stage, with a 5-year survival rate of merely 47.4% (3). Future estimations suggest a continued escalation in the burden of OC in China over the next decade, exceeding global trends (6). This high prevalence highlights the pressing need to comprehend the underlying mechanisms of cancer progression. Furthermore, aberrant lipid metabolism has emerged as a significant risk factor for OC development (7). Unveiling the intricate relationship between lipid metabolism and OC, offering novel insights into its metabolic dysregulation.

Fatty acid reprogramming of cancer cells is crucial for cancer initiation, proliferation and progression (8–10). Lipids, in particular, serve multiple roles: they act as energy reserves for the energetically demanding malignant cells and help alleviate cellular stress during the metastatic process (11, 12). Recent research has shed light on the complex relationship between adipose tissue and the development of OC (1). Adipose tissue is not merely a fat storage depot but also an active endocrine organ secreting various adipokine and inflammatory (13, 14). These cytokines can induce a state of chronic low-grade inflammation and influence cancer metabolism (15), thus establishing a favorable environment for tumorigenesis. Specifically, increased concentrations of circulating free fatty acids have been linked to facilitate cancer cell proliferation (16). Recent studies have identified distinct lipidomic profiles in the blood of ovarian cancer patients compared to healthy individuals, including elevated concentrations of certain fatty acids, phospholipids, and triglycerides (17). These metabolic rewirings are essential for cancer cell membrane formation and signaling pathways and may serve as potential biomarkers for early OC detection and monitoring (18). Consequently, the metabolic cross-talk between adipose and OC tissues through the bloodstream profoundly affects OC progression.

Nevertheless, the majority of metabolomic profiling studies in OC have focused on plasma samples, with limited exploration of OC tissue and no investigation into adipose tissues (19). Lower levels of polyunsaturated fatty acids (PUFAs) like docosahexaenoic acid (DHA) and elevated levels of saturated fatty acids (SFAs) in plasma have been associated with poor prognoses in ovarian cancer patients (20). Specific fatty acids such as DHA and myristoleic acid have been found in higher concentrations in the plasma of OC patients, potentially facilitating cancer growth (21). In ovarian tissues, increased levels of fatty acids such as eicosapentaenoic acid (EPA) and linoleic acid also suggest their roles in cancer cell signaling pathways including inflammation, cellular proliferation, and metastasis, which contributing to tumorigenesis and disease progression (22). Regardless of the progress of research on lipid metabolism in OC, there remains a significant gap in understanding the specific fatty acid profiles across different tissue types within the same individual.

Therefore, Our study aims to bridge this gap by comprehensively analyzing fatty acid profiling in ovarian tissue, subcutaneous fat tissue, and blood between OC and NC patients. By comparing these distinct yet interconnected lipidomic landscapes, we could identify common significant fatty acid level fluctuation in the OC group, offering novel insights into their roles in OC progression, elucidating the metabolic reprogramming of fatty acids and potentially unveiling new biomarkers for early detection and therapeutic targets to improve patient outcomes.

## 2 Materials and methods

### 2.1 Participants and clinical information collection

Participants meeting specific inclusion and exclusion criteria were enrolled as follows: The control group (n=29) including uterine fibroids or endometrioma without any ovarian lesion, while the OC group (n=16) comprised patients diagnosed preoperatively based on serum markers carbohydrate antigen 125 (CA125 > 35 U/mL) and human epididymis 4 protein (HE4 > 70 pmol/L) in pre-menopausal patients, or HE4 >140 pmol/L in post-menopausal patients, along with sonographic evaluation of an adnexal mass. Final diagnoses were confirmed via postoperative pathological examination. Moreover, individuals with severe chronic diseases such as hypertension, diabetes, infectious diseases, or metabolic disorders, as well as those diagnosed with malignancies other than ovarian cancer, were excluded to mitigate recruitment bias. No therapeutic interventions, including chemotherapy, radiotherapy, or surgery, were administered to any of the OC patients. Clinical characteristics, including demographic factors (age and BMI), obstetrical and gynecological history (gravidity and parity), and pathological information (FIGO staging, pathology type, CA125, HE4), were collected at enrollment. The study was ethically approved by the Research Ethics Committee of the Second Affiliated Hospital of Chongqing Medical University, China (202164), and works in accordance with the Declaration of Helsinki. All participants were recruited from the Second Affiliated Hospital of Chongqing Medical University and signed the informed consent before enrolment from July 2020 to June 2021. The patient survival was followed up to 3 years, and Progression-free survival (PFS) was measured. PFS was calculated using the Kaplan-Meier product-limit method from the date of the first day of treatment until the progression of the disease for any cause or for disease.

### 2.2 Specimen collection

Ovarian cancer tissue and subcutaneous fat tissue samples were obtained during surgical procedures. Plasma and mid-stream morning urine samples were collected on the same day prior to surgery. Whole blood was drawn into ethylenediaminetetraacetic acid (EDTA)-coated tubes by trained nurse, followed by centrifugation at 2300 g (10 min, 4°C) with subsequent aliquoting of supernatants into 1.5 ml cryovials. Immediately following collection, all samples were promptly frozen in liquid nitrogen within thirty minutes and subsequently stored long-term at -80°C prior to mass spectrometry analysis. Each sample was assigned a unique laboratory identification number, ensuring sequential processing and preventing laboratory personnel from identifying sample origins.

### 2.3 Sample preparation and fatty acid analysis using gas chromatography-hydrogen flame ionization detector (GC-FID)

#### 2.3.1 Reagents and calibration standard solutions

Fifty-two fatty acid standards were purchased from Nu-CHek PREP, INC. Methanol (HPLC, 99.9%), n-hexane (HPLC, 99.0%), acetyl chloride (99.0%), and potassium carbonate were obtained from Adamas Reagent (Aladdin, China). The internal standard working solution was a 20 mg/L d27-myristic acid mixture solution (Agilent Technologies, USA).

#### 2.3.2 Sample preparation

Tissue samples were weighing 10.00 ± 0.20 mg were dissected into new tubes. Internal standards (5 mL, d27-myristic acid mixture solution) and 400 mL cold methanolic acetyl chloride (10% in methanol) were added. The TissueLyser adapter was pre-cooled at 4°C for 6 hours before tissue homogenization. Subsequently, the tissues were homogenized for two cylcles of 30 second using the TissueLyser II (QIAGEN, USA). Subsequently, all homogenized samples were transferred into a sealed 10 ml glass test tubes. 1600 ul of methanolic acetyl chloride and 300 µL of n-hexane were added, followed by incubation at 95 °C for 1 h. After cooling, 2 ml of 6% potassium carbonate solution was added to enhance separation. After vortexing and centrifugation at 3000 rpm for 10 min, 100 µL of the upper organic phase was collected for the GC-FID analysis.

#### 2.3.3 GC-FID analysis

The GC-FID analysis was conducted using an Agilent 7890B gas chromatograph (Agilent Technologies, USA). A 1 μL aliquot of the upper organ phase (fatty acid methyl ester) was injected into the GC inlet in a 1:2 split mode. Nitrogen served as the carrier gas, maintaining a constant flow rate of 1 mL/min to facilitate the separation of fatty acids. The separation was performed using the DB-fast FAME capillary column (30 m×0.25 mm×0.25 μm, Agilent Technologies, USA), with the following an oven temperature program: (1) 80°C hold for 0.5 minutes; (2) ramped to 165°C at 40°C/min; (3) increased to 230°C at 4°C/min; and (4) hold at 230°C for 4 minutes. The FID temperature remained constant at 260°C, with air and hydrogen flow rates set at 400 mL/min and 40 mL/min, respectively. Additionally, nitrogen was utilized as the make-up gas at a constant flow rate of 25 mL/min.

### 2.4 Quality control (QC)

QC samples were prepared by combining 1.00 ± 0.10 mg of each respective sample into a new tube, following the methodology outlined in section 3.2. The acquisition of a QC spectrum was conducted at intervals for every 15 samples.

### 2.5 Fatty acid identification, extraction, normalization and quantification

Fatty acids were identified based on their predetermined retention times, employing 52 fatty acid chemical standards within the same batch. The relative concentrations of fatty acids were assessed by measuring chromatographic peak areas corresponding to the identified fatty acids. Background contamination and carryover effects were mitigated by subtracting the values obtained from blank samples. To ensure quantitative accuracy, the relative concentrations of identified fatty acids were initially normalized using the internal standard (d27-myristic acid). Biomass correction was achieved by the weight of the samples. Individual fatty acid concentrations were quantified using calibration curves derived from corresponding chemical standards, ranging from 0 to 273.12 µg/ml.

### 2.6 RT-qPCR

Ovarian tissues were homogenized by mechanical fragmentation followed by sonication. Total RNA was isolated using Trizol reagent (Invitrogen/Thermo Fisher Scientific) according to the manufacturer’s instructions. The extracted RNA was transcribed into cDNA and underwent RT-qPCR (TaKaRa). Each sample was assayed in technical triplicates to ensure reproducibility. Specific primer sequences for each gene were shown in the following: Fatty Acid Synthase (*FASN*, forward: TAC CTG AGC ATA GTG TGG AAGAC; reverse: GGT ACA CCT TCC CAC TCA CTAC), elongation of very long chain fatty acids protein 1 (*ELOVL1*, forward: GTC TAC AAC TTC TCA CTG GTG GC; reverse: AAG TGC CTC AGG GCT GTT GGAA), elongation of very long chain fatty acids protein 2 (*ELOVL2*, forward: TCC ACT TGG GAA GGA GGC TACA; reverse: CCA GGA ACT CTA CTG ATT TGG AG), elongation of very long chain fatty acids protein 5 (*ELOVL5*, forward: ACG TCT ACC ACC ATG CCT CGAT; reverse: TGG AAG GGA CTG ACG ACA AACC), elongation of very long chain fatty acids protein 6 (*ELOVL6*, forward: CCA TCC AAT GGA TGC AGG AAA AC; reverse: CCA GAG CAC TAA TGG CTT CCTC), elongation of very long chain fatty acids protein 7 (*ELOVL7*, forward: CCT ACT ATG GAC TTT CTG CAT TGG; reverse: GAA CTG GCT TAT GTG GAT GGCG), Fatty acid desaturase 1 (*FADS1*, forward: CTG TCG GTC TTC AGC ACC TCAA; reverse: CTG GGT CTT TGC GGA AGC AGTT), Fatty acid desaturase 2 (*FADS2*, forward: CTG GTT CAG TGG ACA CCT TAA CT; reverse: AGT AGC GGC TTC TCC TGG TAT TC), and *GAPDH* (forward: GCT CTC TGC TCC TCC TGT TC; reverse: CGA CCA AAT CCG TTG ACT CC). *GAPDH* was used to normalize the expression level. The expression values were log2 transformed, and the relative gene expression levels were calculated using the comparative ΔΔCT method.

### 2.7 Statistical analysis

Statistical analyses were conducted using R programming language. Student’s t-test and non-parametric Mann-Whitney U test were employed to compare clinical characteristics between the NC and OC groups. Chi-square test was utilized for pairwise comparisons of categorical variables such as gravidity and parity. Partial least squares discriminant analysis (PLS-DA) was performed using R to visualize the discrimination between groups. To account for confounding factors, particularly differences in patient BMI, binary logistic regression was employed to confirm differences in fatty acid concentration between the NC and OC groups. False discovery rates (FDR) were calculated for metabolites using the q-value R package (23), P-value< 0.05 and corresponding FDR< 0.2 was considered statistically significant. UpSet diagram and heatmap were completed by UpSetR and ggplot2 R packages respectively (24, 25). Advanced volcano plots were created using the OmicStudio tools at https://www.omicstudio.cn/tool. To assess the diagnostic ability, receiver operating characteristic (ROC) curve analysis was conducted and the, Area Under The Curve (AUC) was calculated. Logistic regression analysis was used to assess the impact of the significant fatty acids on the response rate, and the results were reported as an odds ratio (OR) with 95% CI. Metabolic pathways were predicted using the KEGG database, and chord plots connecting metabolites and their participating pathways were reconstructed using the GOplot R package. All statistical analyses were performed using the R program v4.0.3 (26).

## 3 Results

### 3.1 Clinical characteristics of study participants

In this study, we conducted a meticulous analysis of the clinical characteristics of 45 participants, comprising 29 individuals in the control group and 16 diagnosed with ovarian cancer (**Table 1**). The FIGO criteria, the proportions across stages I, II, III, and IV were 31.25%, 12.50%, 50.00%, and 6.25%, respectively. The ovarian cancer-specific biomarkers, namely CA125 and HE4, exhibited substantially higher levels in the ovarian cancer group compared to the control group (p<0.0001). No statistically significant disparities were observed in age, gravidity, and parity between the control and ovarian cancer groups, except for BMI (p=0.042), which will be adjusted through logistic regression.

**Table 1:** Clinical characteristics of study participants.

Characteristics	Control (n=29)	Ovarian cancer (n=16)	P-value
Age, a(b), years	50.00 (36.00, 54.00)	49.50 (46.25, 57.00)	0.456
BMI, a(b), kg/m^2^	23.56 (21.27, 26.04)	20.42 (19.98, 24.56)	0.042
Gravidity, a(b)	2 (1.5, 3.0)	3 (2.0, 4.0)	0.714
Parity, a(b)	1 (1, 1)	1 (1, 1)	0.758
FIGO staging			
I, n (%)	n/a	5 (31.25%)	
II, n (%)	n/a	2 (12.50%)	
III, n (%)	n/a	8 (50.00%)	
IV, n (%)	n/a	1 (6.25%)	
Pathology type			
high-grade serous ovarian cancer, n (%)	n/a	11 (68.75%)	
Mucinous, n (%)	n/a	1 (6.25%)	
Endometrioid, n (%)	n/a	1 (6.25%)	
Yolk Sac Tumor, n (%)	n/a	1 (6.25%)	
Papillary serous carcinoma, n (%)	n/a	1 (6.25%)	
Low-grade serous carcinoma, n (%)	n/a	1 (6.25%)	
CA125, a(b), U/ml	21.35 (12.60, 44.88)	641.00 (144.50, 1000.00)	0.0001
HE4, a(b), pmol/l	37.00 (34.60, 45.40)	169.00 (52.40, 659.50)	0.0001

a, median; b, confidence interval (25th percentile, 75th percentile; n, numbers; n/a, not applicable; CA125, cancer antigen 125; HE4, human epididymis protein.

### 3.2 The specific metabolomic profiles of subcutaneous fat tissue, plasma and ovarian tissue between OC and NC groups

**Figure 1:**
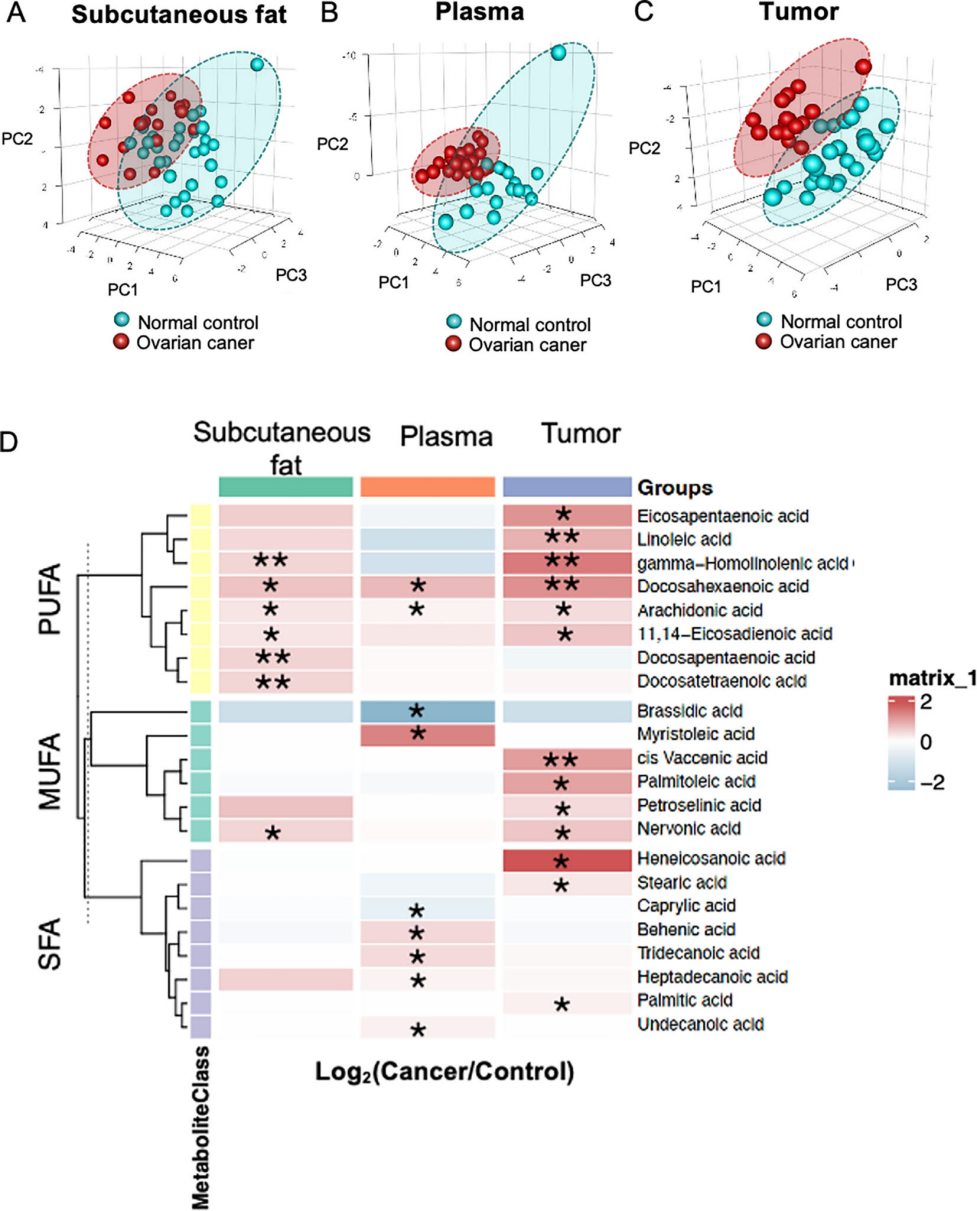
Fatty acid profiles. Partial least squares discriminant analysis (PLS-DA) of metabolites in **(A)** subcutaneous fat tissue, **(B)** plasma, and **(C)** ovarian tissue. **(D)** The heatmap displays the ratio of metabolite levels detected in each group. Red and blue colors denote higher and lower metabolite concentrations in cancer groups compared to normal groups respectively (*p< 0.05 and **p<0.01).

A PLS-DA was established using all identified metabolites from samples of subcutaneous fat tissue, plasma and ovarian tissue samples. The PLS-DA of subcutaneous fat tissue and plasma samples showed a greater overlap in the confidence interval areas (**Figures 1A, B**), while ovarian tissue samples showed a distinct difference between cancer and control groups, with evident separation (**Figure 1C**). The PLS-DA model validation metrics for tumor tissue, subcutaneous fat, and blood samples are shown in **Supplementary Figure 1**. While the tumor tissue model demonstrated optimal predictive performance (Q² > 0.5, permutation test p < 0.05), the subcutaneous fat and plasma models exhibited inferior validation results, with low Q² values (plasma: Q² < 0.4; subcutaneous fat: Q² < 0, indicating no predictive utility).

Ovarian tissue had a higher number of significant metabolites (n=13), whereas subcutaneous fat tissue (n=7) and plasma (n=9) detected fewer significant metabolites respectively. A heatmap revealed significant alternations in twenty-two metabolites between OC and NC groups in the subcutaneous fat tissue, plasma and ovarian tissue samples (**Figure 1D**) (p<0.05, q<0.2). The majority of different metabolites were PUFAs. Most fatty acids exhibited relatively higher concentrations in the OC group compared to the NC group.

### 3.3 Comparative analysis of fatty acid profiles among subcutaneous fat tissue, plasma and ovarian tissue.

**Figure 2:**
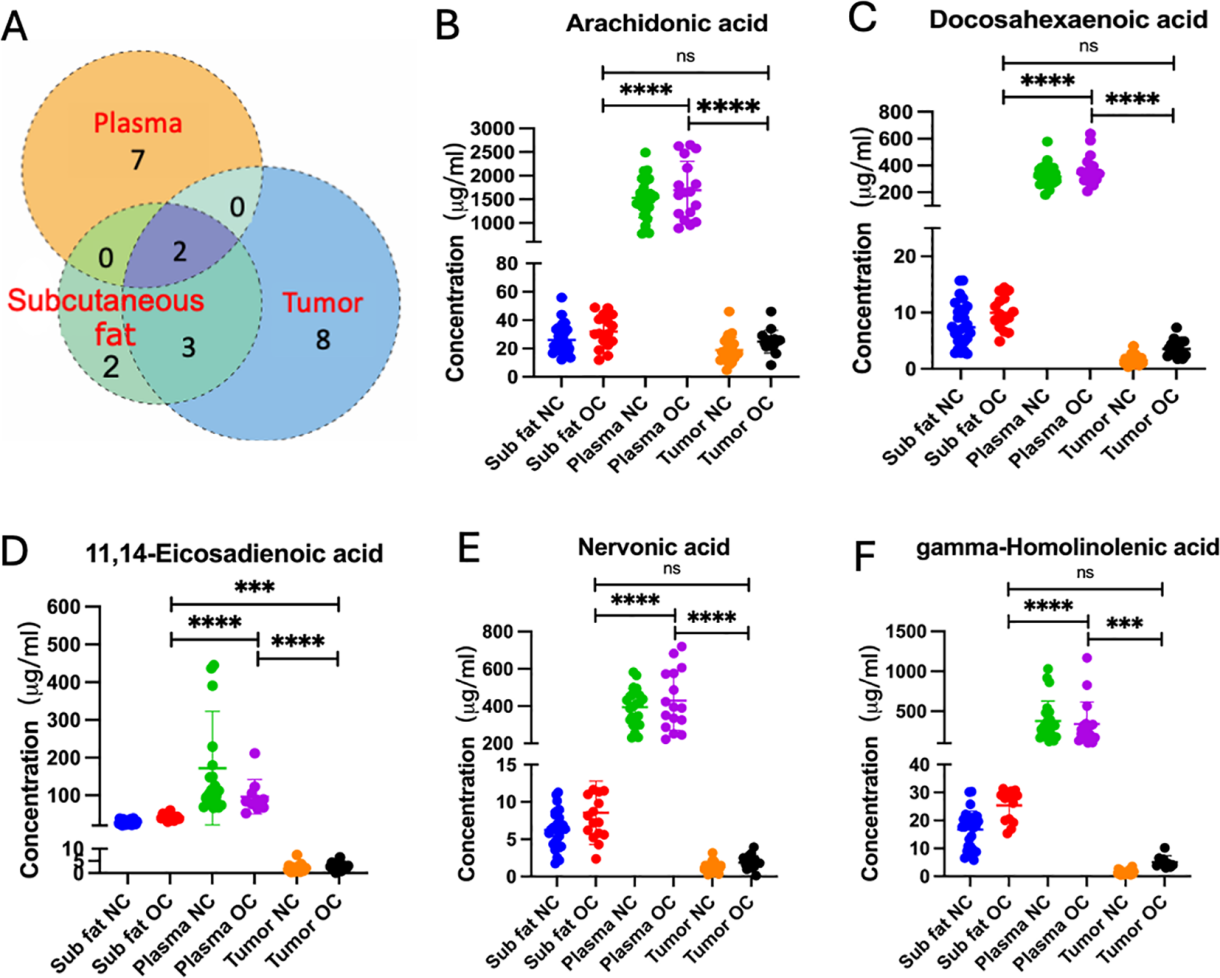
**(A)** Venn diagram of differential (p < 0.05) metabolites. The individual or connected dots represent the unique or shared intersections of metabolites across subcutaneous fat, plasma, and ovarian tissue samples. Scatter plots depicting the concentrations of specific fatty acids, including **(B)** arachidonic acid, **(C)** docosahexaenoic acid, **(D)** 11,14-eicosadienoic acid, **(E)** nervonic acid, and **(F)** gamma-homolinolenic acid, across subcutaneous fat, plasma, and tumor tissues in normal control (NC) and ovarian cancer (OC) groups. Statistical significance is indicated by asterisks: ns, not significant, *** (p < 0.001), and **** (p < 0.0001).

To eliminate the potential confounding effects of BMI, logistic regressions were performed to compare metabolite changes between NC and OC patients. The Venn diagram indicates distinct patterns of metabolite convergence and divergence across various tissue types. Specifically, seven, eight, and two unique metabolites were identified in plasma, ovarian tumor tissue, and subcutaneous fat respectively. Furthermore, three metabolites were shared between subcutaneous fat and tumor tissue, while only two metabolites were common among subcutaneous fat, plasma, and ovarian tissues (**Figure 2A**). These commonly shared metabolites may serve as robust biomarkers for ovarian cancer detection and monitoring. Detailed comparisons of fatty acid concentrations (**Figures 2B–F**) revealed significant variations across different sample types in NC and OC groups. AA and DHA were significantly different between NC and OC across all three sample types and found at higher plasma concentrations than the other sample types, while both PUFAs displayed similar concentrations between subcutaneous and tumor tissues (**Figures 2B, C**). Three PUFAs, including 11,14-eicosadienoic acid, nervonic acid and gamma-homolinolenic acid (GHLA), were only significantly different between OC and NC groups in subcutaneous fat and tumor tissue, with the highest concentration found in the plasma samples (**Figures 2D–F**). The common significant changes in fatty acid concentration observed across different specimen types further highlight the importance of fatty acid metabolism in OC pathogenesis.

### 3.4 Biomarker discovery and risk factors

**Figure 3:**
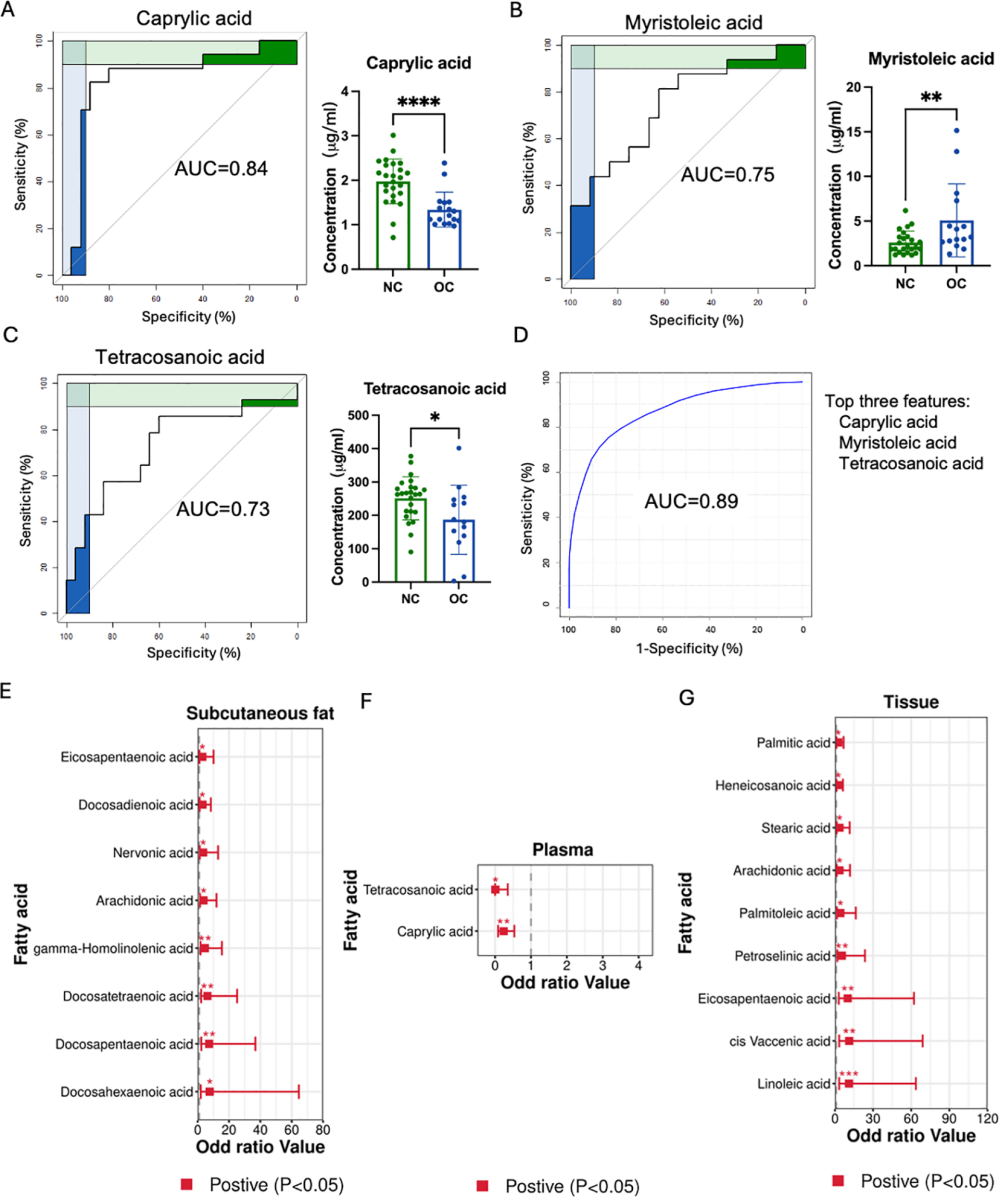
Biomarker screening and OC risk. **(A–C)** Three metabolites with an area under the curve (AUC) greater than 0.7 in the ovarian cancer tissue. **(D)** Combining all three shortlisted fatty acids in the ROC analysis. The metabolites with odds ratios excess than one or less than one in subcutaneous fat **(E)**, plasma **(F)**, and ovarian cancer **(G)** tissues. (*p < 0.05, ** p < 0.01, *** p < 0.001, **** p < 0.0001).

ROC curves were employed to evaluate metabolites as potential biomarkers for OC. In plasma sample (**Figures 3A–C**), three metabolites with an area under the ROC curve exceeding 0.73 were identified, whereas no promising AUC result was observed in subcutaneous fat or ovarian tissue. **Figure 3A** showed caprylic acid exhibited the highest sensitivity and specificity, with an AUC of 0.84. **Figures 3B, C** displayed that myristoleic acid and tetracosaenoic acid also demonstrated good sensitivity and specificity, with AUC values over 0.70. Combining all three shortlisted fatty acids in the ROC analysis illustrated higher sensitivity and specificity, achieving an AUC of 0.89 (**Figure 3D**).

Furthermore, the odds ratio (OR) was employed to evaluate the association between OC risk and identified metabolites. Eight and nine metabolites with OR greater than one were found in subcutaneous fat and ovarian tissues respectively, while two metabolites had an OR less than one. Specifically, eicosapentaenoic acid, docosadienoic acid, nervonic acid, AA, GHLA, docosatetraenoic acid (DTA), docosapentaenoic acid (DPA), and DHA were associated with higher OC risk in the subcutaneous fat group (**Figure 3E**). In the ovarian tissue, palmitic acid, heneicosanoic acid, stearic acid, arachidonic acid, palmitoleic acid, petroselinic acid, eicosapentaenoic acid, cis-vaccenic acid and linoleic acid showed higher OC risk (**Figure 3G**). Meanwhile, tetracosaenoic acid and caprylic acid were related to lower OC risk in the blood sample (**Figure 3F**). Thus, these highlighted metabolites could potentially serve as biomarkers and indicate the presence of risk factors associated with the development of OC.

### 3.5 Association of unsaturated fatty acid reprograming with BMI, FIGO stage, recurrence, and prognosis in OC patients

**Figure 4:**
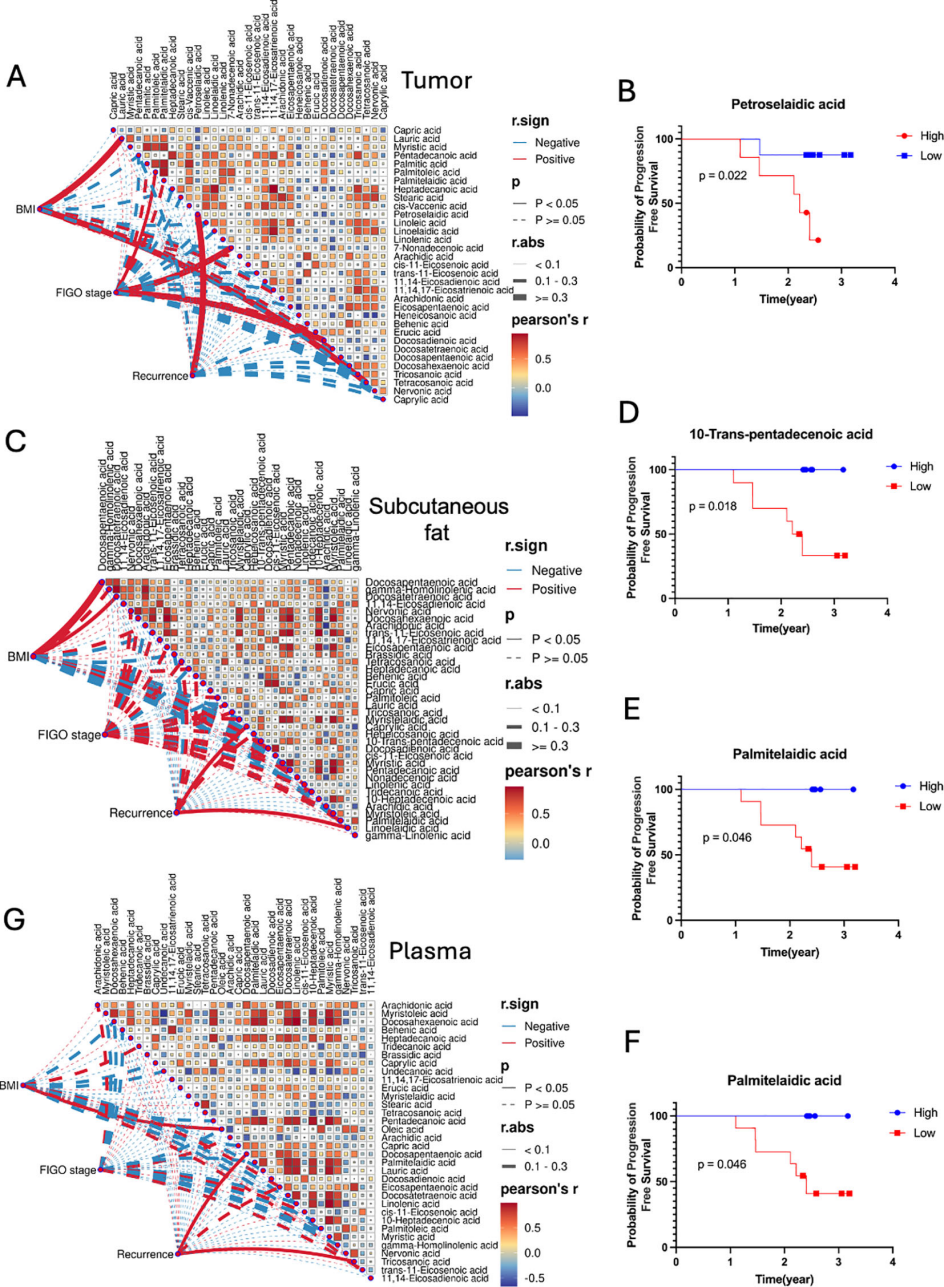
Correlation matrices between fatty acids, BMI, FIGO stage and Recurrence for **(A)** tumor, **(C)** subcutaneous fat, and **(G)** plasma samples. Pearson’s r color gradient from blue to red represents the correlation values from -1 to 1. Mantel’s r.sign indicates whether the correlation is positive or negative. Mantel’s p displays the statistical significance of the correlations, with p-values less than 0.05 considered significant. Mantel’s r.abs represents the absolute value of the correlation, with different line thicknesses indicating the strength of the correlation (thin for <0.1, medium for 0.1-0.3, thick for >0.3). Kaplan-Meier Survival Curves of fatty acids for Tumor **(B)** and Subcutaneous fat **(D–F)**.

Our study investigated the correlation between fatty acid levels, BMI, FIGO stage and recurrence (recurrence/non-recurrence) across tumor tissue, subcutaneous fat, and plasma samples (**Figure 4**). In tumor tissues samples, petroselaidic acid exhibited a positive and statistically significant correlation with the Recurrence (**Figure 4A**), with higher levels of this fatty acid are associated with a poorer prognosis (**Figure 4B**). Additionally, lauric acid and tetracosanoic acid were positively correlated with BMI in tumor tissue, while palmitic acid, 7-Nonadecenoic acid, and docosadienoic acid were positively correlated with FIGO stage in tumor tissue (**Figure 4A**). In the subcutaneous fat tissue, caprylic acid and linoelaidic acid were positively correlated with the Recurrence, while BMI showed a positive correlation with docosapentaenoic and docosatetraenoic acids, there was no significant correlation between FIGO stage and subcutaneous fat tissue (**Figure 4C**). Furthermore, Lower levels of 10-trans-pentadecenoic acid (**Figure 4D**) and palmitelaidic acid (**Figure 4E**) were also associated with poorer prognosis. Higher levels of linoelaidic acid were associated with a worse prognosis (**Figure 4F**). In plasma (**Figure 4G**), DPA and tricosanoic acid levels were positively correlated with the Recurrence, Oleic acid shows positively correlated with the BMI, while no significant correlations were found between FIGO stage and any fatty acids, nor were significant survival correlations observed. These findings suggest that fatty acid levels may influence prognosis differently across tissue types.

### 3.6 Metabolic pathways enrichment analysis of ovarian cancer

**Figure 5:**
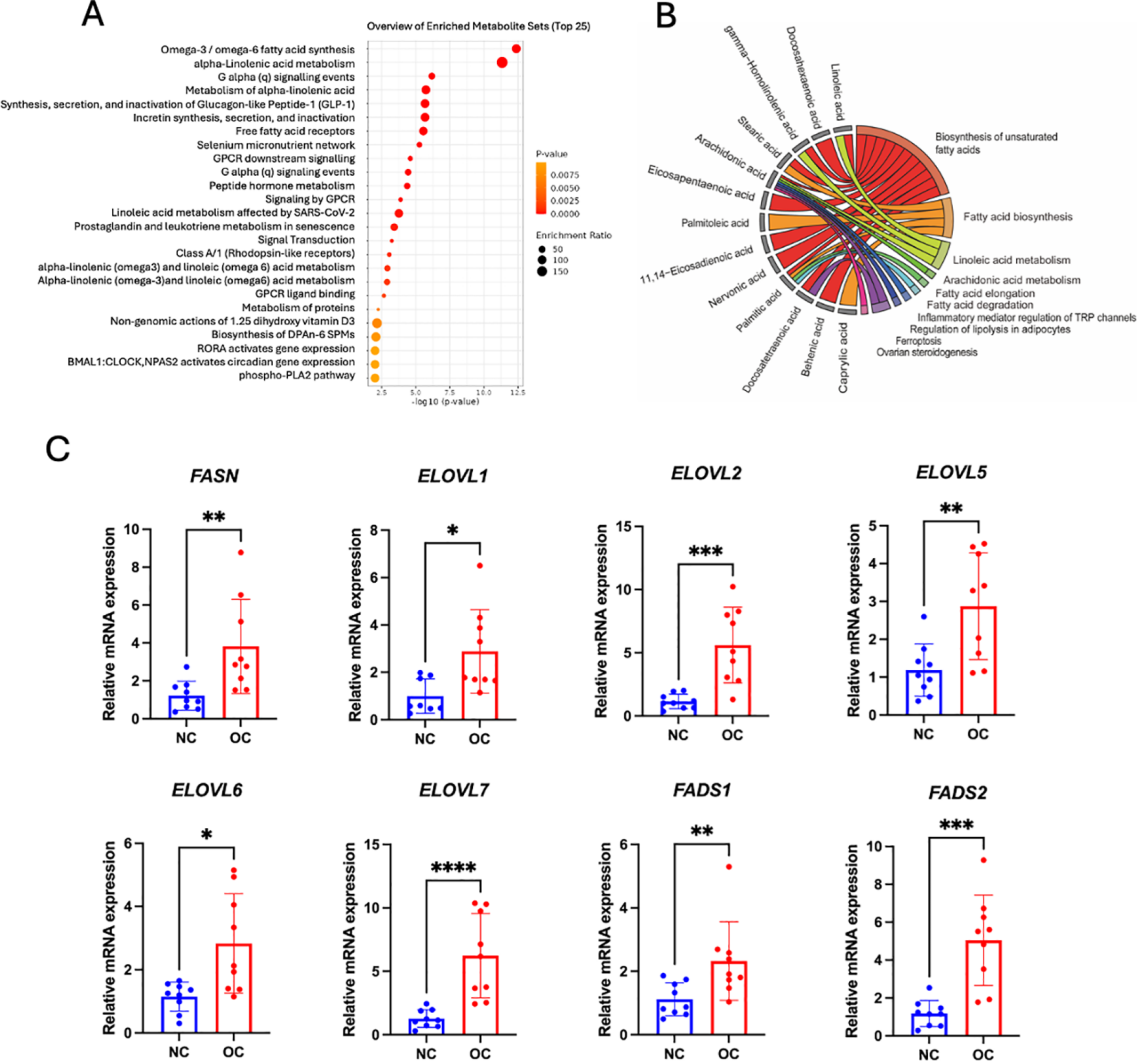
Metabolic pathway analysis. **(A)** Fatty acid enrichment overview through graphs, showing pathway enrichment levels with a red-to-yellow gradient denoting significance, from highly significant (red) to less significant (yellow). **(B)** Chord plot depicting metabolite connections with pathways: red (biosynthesis of unsaturated fatty acids), orange (fatty acid biosynthesis), light green (linoleic acid metabolism), dark green (arachidonic acid metabolism and fatty acid elongation), cyan blue (fatty acid degradation), blue (inflammatory mediator regulation of TRP channels), royal blue (regulation of lipolysis in adipocytes), purple (ferroptosis), and pink (ovarian steroidogenesis). **(C)** Bar and dot plots showing the relative mRNA expression levels of key lipid metabolism enzymes, including fatty acid synthase (*FASN*); elongation of very long chain fatty acids proteins (*ELOVL1*, *ELOVL2*, *ELOVL5*, *ELOVL6*, and *ELOVL7*); and fatty acid desaturases (*FADS1* and *FADS2*) in control and cancer groups (n = 9 biological replicates per group). *p < 0.05, ** p < 0.01,*** p < 0.001, **** p < 0.0001.

To elucidate the underyling metabolic reprogramming in response to OC, the top 25 enriched pathways are displayed in **Figure 5A**, with significant metabolites annotated in each KEGG pathway presented in **Figure 5B**. Most of the metabolites in ovarian tissue, subcutaneous fat tissue and plasma were involved in the highest enrichment pathways such as omega-3/omega-6 fatty acid synthesis, alpha-linolenic acid metabolism, which highlight the importance fatty acid dysregulation in tumor growth and progression.

### 3.7 Expressions of enzymatic genes related to fatty acid synthesis, elongation and desaturation

To validate our enrichment pathway related to fatty acid dysregulation in tumors, we performed RT-qPCR to quantify the expression of key genes involved in fatty acid synthesis (*FASN*), elongation (*ELOVL1*, *ELOVL2, ELOVL5*, *ELOVL6* and *ELOVL7*), and desaturation (*FADS1* and *FADS2*). **Figure 5C** shows that all these genes had expressed a higher level of ovarian tissue mRNA in the cancer group compared to the control group. These results further supported that shortlisted fatty acid metabolic pathways were likely more active in ovarian cancer patients.

## 4 Discussion

Fatty acid reprogramming is reported to reflect the metabolic demands related to ovarian cancer progression. This study explored the fatty acid profilings of plasma, tumor and subcutaneous fat tissues from a cohort of sixteen OC patients and twenty-nine NC patients to investigate the underlying modulations in fatty acid composition. Our findings indicated that distinct metabolite profiles distinguish OC from NC groups across all three sample types. Specifically, DHA and AA exhibited elevated levels in OC patients across subcutaneous fat, plasma and ovarian tissue samples. Notably, subcutaneous fat displayed an elevated concentration of AA and tetracosanoic acid, which are crucial in regulating cell death and inflammatory mediator responses. Plasma samples, in particular, exhibited the highest concentrations of caprylic acid among different tissues, with an AUC of 0.85, suggesting its potential as a biomarker for ovarian cancer. Furthermore, four fatty acids (petroselaidic acid, 10-trans-pentadecenoic acid, palmitelaidic acid, and linoelaidic acid) were found to be associated with progression-free survival. By unraveling the intricate metabolic mechanisms underlying these fatty acid modulations in OC patients, this study offers valuable insights that could contribute to our understanding of ovarian cancer progression.

### 4.1 Fatty acid reprogramming in OC

This study is the first to delineate distinct fatty acid metabolism across subcutaneous fat, plasma, and ovarian tissue in OC patients. Our findings suggest that OC potentially induces systemic metabolic reprogramming, altering adipocyte function to support tumor growth. Notably, significant alterations in fatty acid composition were observed in subcutaneous fat from OC patients. All significant PUFAs (**Figure 1D**), including AA, GHLA, DTA, DPA, and DHA, were elevated in the OC group. This was accompanied by the upregulation of key enzymes involved in synthesis (*FASN*), elongation (*ELOVL1*,*2*,*5*,*6*,and *7*), and desaturation (*FADS1* and *FADS2*) in ovarian tissue compared to NC groups (**Figure 5C**), implicating these pathways in cancer progression (27). These findings align with previous studies, as AA, GHLA, and DPA have been shown to elevate cancer risk in subcutaneous fat (28, 29). Furthermore, Frankhouser et al. indicated that DHA and EPA might promote cancer progression through inflammatory cascades (30) despite DHA also being thought to have antitumorigenic beneficial effects for OC treatment and prevention (31). Some researchers suggest that DHA and EPA may have dual effects on health, depending on their levels and the condition of the patient (32). We also found that a high level of linoelaidic acid was associated with poorer progression-free survival in subcutaneous fat (**Figure 4**). This is consistent with findings by Helene et al, who reported a positive and independent association between adipose tissue and recurrence-free survival (20). Moreover, Previous research suggests that linoelaidic acid can further exacerbate cancer progression by increasing oxidative stress and inflammation in subcutaneous fat, and it is also relative to the modulation of ferroptosis, a mechanism that cancer cells exploit to evade cell death (33, 34). Moreover, PUFAs, as a distinct functional lipid class, are dynamically regulated during cell-state transitions and influence OC susceptibility to ferroptosis (35). In addition, our study provides compelling evidence linking alterations in the biosynthesis of n-6 fatty acids to the development of OC, with particular emphasis on AA and PA. Through meticulous analysis of metabolomic profiles in ovarian tissue, we observed significantly elevated levels of LA, AA and PA in OC patients compared to NC patients. Interestingly, the role of LA in tumor progression remains controversial. Several studies have reported anti-cancer effects of LA, including the induction of cell cycle arrest and modulation of key signaling pathways (36). However, other evidence suggests a pro-tumorigenic role in specific contexts (37, 38). For instance, a recent study published in Science demonstrated that LA promotes the growth of triple-negative breast cancer by activating the FABP5-mTORC1 signaling axis (39). These findings support the concept that dysregulated fatty acid metabolism contributes to OC progression. Other studies have also reported various fatty acids or metabolic pathways in OC development. For instance, some research suggests that n3 fatty acids may have protective effects against OC (40). Thus, such divergent findings underscore the complexity of lipid metabolism in ovarian cancer biology.

**Figure 6:**
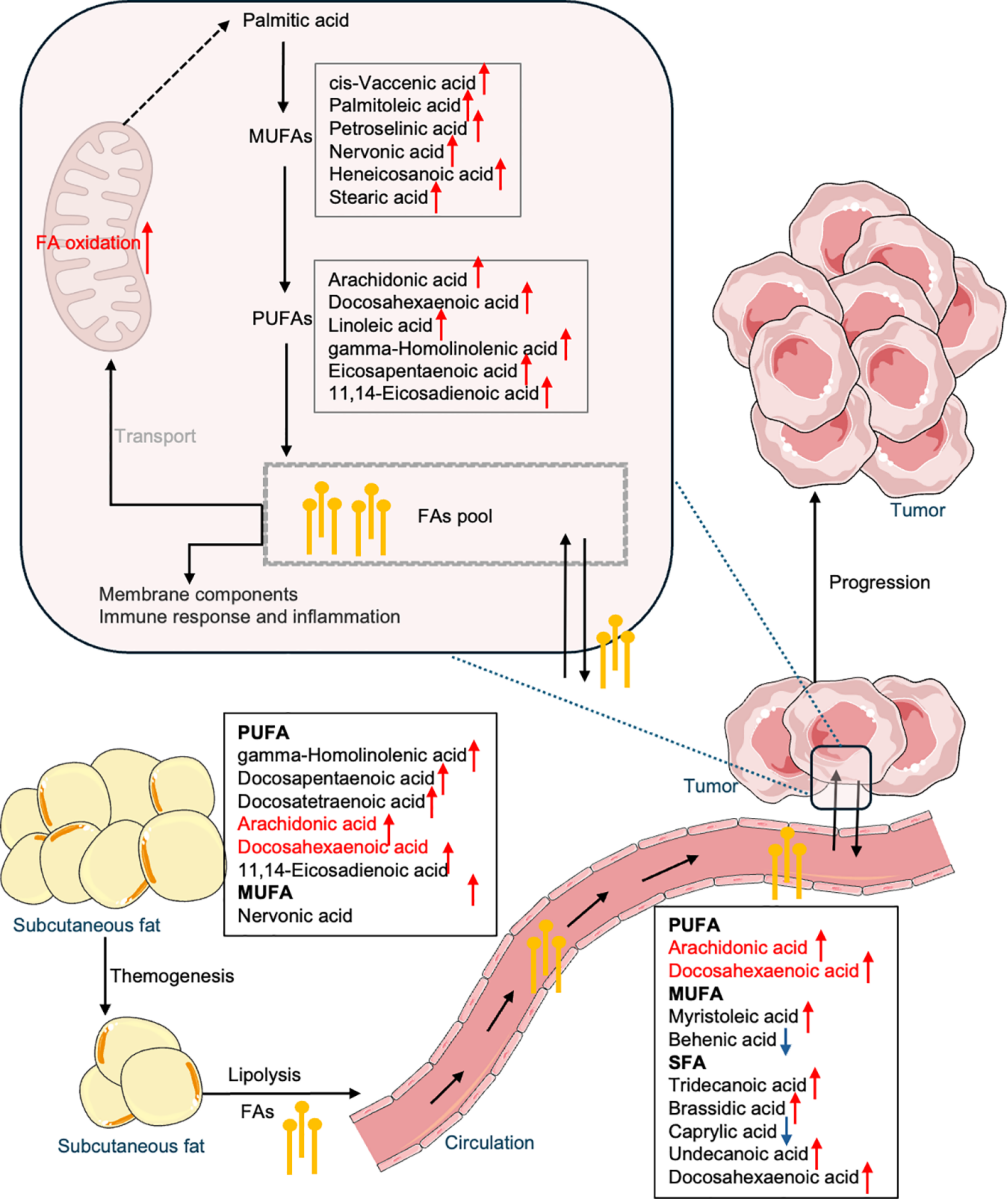
Summary of fatty acid reprogramming across subcutaneous fat, blood, and OC tissue.

Therefore, the upregulation of fatty acid metabolism may contribute to thermogenesis in OC patients, invariably associated with lipid catabolism and mobilization, a hallmark of cancer-associated cachexia (41, 42). Increased lipolysis enhances lipid mobilization of WAT, significantly reducing adipose depots (43). Fatty acids released from lipolysis enter the circulation and undergo alterations in availability and composition, becoming a crucial source of energy and building blocks for the tumor. These fatty acids and those synthesized *de novo* by the cancer cells constitute a fatty acid pool that serves as a centralized resource. This pool supplies essential components for membrane synthesis, signaling, and β-oxidation for energy production, thereby sustaining tumor growth and progression. This metabolic reprogramming highlights how changes in fatty acid profile within subcutaneous adipose tissue are intricately linked to the metabolic needs of the tumor, illustrating a network (**Figure 6**) through subcutaneous fat, plasma, and ovarian tissue that supports cancer cell growth, survival, and progression.

### 4.2 Biomarker implication

Recent research has highlighted the importance of plasma fatty acids as potential biomarkers for cancer diagnosis due to their discriminatory properties (44). Specifically, saturated fatty acids (SFAs) have been associated with reduced cancer risk (35, 44). In our study, two SFAs (caprylic acid and tetracosanoic acid) were identified as contributing to this protective effect. We specifically identified plasma short-chain fatty acids (SCFAs), such as caprylic acid and tetracosanoic acid, as promising biomarkers for OC due to their notable discriminatory power, as demonstrated in **Figure 3A**. This is consistent with previous findings that highlight caprylic acid’s inhibitory effects on various cancer cells (45). Similarly, tetracosanoic acid, despite its rare association with OC, has been reported to protect against cancer progression, necessitating further investigation (46). These findings underscore the potential of plasma fatty acid profiling as a predictive tool for cancer biomarker discovery.

## 5 Limitations

While our study provides valuable insights into the role of fatty acid metabolism in OC, several limitations warrant consideration. The sample size is relatively small, which may affect the generalizability of the findings. Future studies with larger cohorts are needed to validate the identified biomarkers and elucidate the mechanisms underlying lipid metabolism dysregulation in OC. Furthermore, whole-genome sequencing of OC should be conducted to identify specific mutation statuses and delineate distinct fatty acid reprogramming patterns across genetic subtypes.

## 6 Conclusion

In conclusion, our study reveals significant differences in fatty acid profiles among OC and NC patients across plasma, tumor and subcutaneous fat tissues. Our findings proposed that plasma capric acid, tetracosanoic acid, and myristoleic acid may serve as potential biomarkers for OC diagnosis. This comprehensive analysis highlights how reprogramming in fatty acid metabolism occurs across plasma, tumor and subcutaneous fat tissues, suggesting interconnected metabolic alterations that contribute to OC progression. Further research is needed to validate these findings and elucidate the mechanistic underpinnings of lipid metabolism dysregulation in OC, thereby facilitating the development of novel therapeutic strategies to improve patient outcomes.

## Data Availability

The raw data supporting the conclusions of this article will be made available by the authors, without undue reservation.

## Supplementary material

Supplementary Figure 1 Model validation metrics for PLS-DA analysis. Histogram of R^2^ (variance explained) and Q^2^ (predictive ability) values for tumor tissue **(A)**, plasma **(B)**, and subcutaneous fat **(C)**. Dashed blue, red, and light blue histograms indicate the observed Accuracy, R^2^, and Q^2^, respectively. A red asterisk marks the number of significant components. Distribution of permuted Q^2^ values (blue bars) from 1000 random label shuffles with a deviation from the null distribution for tumor tissue **(D)**, plasma **(E)**, and subcutaneous fat **(F)**.
