# Supplementary figures and images for: Elucidating the role of fatty acid reprogramming in ovarian cancer: insights cross-talk between blood, subcutaneous fat, and ovarian cancer tissues
Source: Front Oncol. 2025 Apr 30;15:1530487. doi: 10.3389/fonc.2025.1530487 (PMC12074969; doi:10.3389/fonc.2025.1530487)

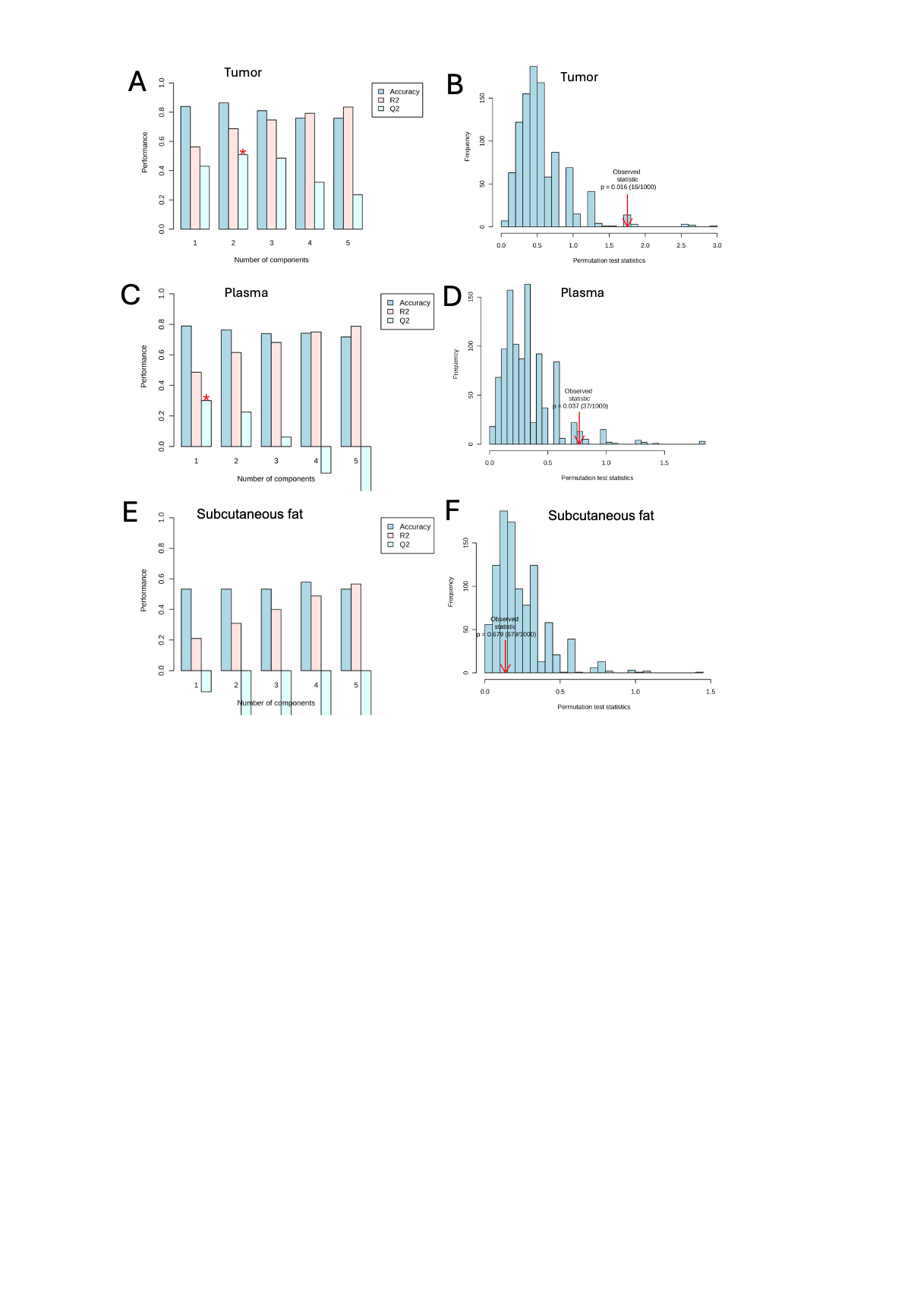

Supplement: Supplementary Figure 1 — Model validation metrics for PLS-DA analysis. Histogram of R2 (variance explained) and Q2 (predictive ability) values for tumor tissue (A), plasma (B), and subcutaneous fat (C). Dashed blue, red, and light blue histograms indicate the observed Accuracy, R2, and Q2, respectively. A red asterisk marks the number of significant components. Distribution of permuted Q2 values (blue bars) from 1000 random label shuffles with a deviation from the null distribution for tumor tissue (D), plasma (E), and subcutaneous fat (F). [file Image1.tiff]
